# AI-Assisted Analysis of Sentiment Changes Among Repeat Users of Digital Peer Support: A Retrospective Longitudinal Study

**DOI:** 10.3390/jcm15197677

**Published:** 2026-10-04

**Authors:** Farbod Sedaghati, Anya Stetsenko, Ilayda Ozsan McMillan, Zara Dana, Harpreet Nagra

**Affiliations:** Supportiv, 2222 Harold Way, Berkeley, CA 94704, USAanya@supportiv.com (A.S.); ilayda@supportiv.com (I.O.M.); zara@supportiv.com (Z.D.)

**Keywords:** emotional well-being, sustained sentiment trajectories, digital peer support, repeated user engagement, mental health, machine learning, artificial intelligence

## Abstract

**Background**: Digital peer support (DPS) is a scalable tool to provide emotional support across diverse user populations. Previous studies were limited to single-session outcomes, while the impact of repeated DPS sessions is not well understood. **Objectives**: This study evaluated sentiment trajectories among repeat users of a 24/7, anonymous, real-time moderated DPS service and examined whether DPS engagement across repeated sessions was associated with changes in negative sentiments (depression, loneliness, despair, grief, and work-related stress). User engagement patterns across different conversation topics, and topic-specific differences in DPS use were also explored. **Methods**: We conducted a retrospective analysis of 13,686 anonymous live DPS chat sessions (January 2023 to December 2024) from 8084 distinct Supportiv DPS users. Of these, 4099 chat sessions from 596 unique repeat users met the predefined inclusion criteria of at least two sessions with a sentiment score ≥5, representing at least moderate expression of the negative sentiment of interest, and a minimum of three scored messages per session. Sessions were categorized into mental health topics using a large language model, GPT-4o-mini. For each topic (depression, grief, loneliness, suicidality, and work-related), relevant sentiment intensity scores were generated by using a few-shot approach based on scale definitions. Individual message-level sentiment trajectories were descriptively characterized for each user using ordinary least squares (OLS) regression across accumulated conversation time. User engagement patterns, session characteristics, and self-reported demographics were compared using nonparametric tests. **Results**: Over the two-year study period of repeat DPS users, among user-level trajectories with statistically significant OLS slopes, the proportion with negative slopes was 90.9% for despair, 86.1% for depression, 85.7% for grief, 82.4% for work-related stress, and 72.7% for loneliness. Approximately 40–55% of all negative trajectories reached statistical significance. Suicidality-related sessions were ~20% longer and shared ~35% more resources, while loneliness sessions included 14% more resources than other topics. Users focusing on loneliness and suicidality had longer return intervals (11.8 and 18.6 days, respectively) compared to the average (10.4 days). **Conclusions**: Analyses showed that some users demonstrated downward message-level sentiment across accumulated conversation time. Given its accessibility and scalability, DPS is a promising mental health support tool. Prospective controlled studies are needed to determine whether repeated DPS use produces sustained benefits.

## 1. Introduction

### 1.1. Mental Health Burden and Scalable Support Shortage

Emotional distress is increasingly prevalent in the United States and globally, with recent surveys indicating that up to one in three adults report problems with their mental health and emotional well-being [1,2,3]. Mental health problems have immense economic costs, with depression and anxiety alone costing the global economy $1 trillion annually in lost productivity [4,5]. Overall mental-health-care-related costs in the United States exceed $280 billion per year through health care spending, disability, and reduced productivity [6,7]. These trends emphasize the need for interventions that address emotional distress at a population level.

Emotional distress includes a range of dimensions, such as depression, loneliness, stress, grief, despair, and anxiety, among others, all of which impair well-being, quality of life, and functioning and increase the risks of serious health problems [8,9,10]. Data from the 2022 Behavioral Risk Factor Surveillance System (BRFSS) indicated that 32.1% of adults experienced loneliness, 24.1% lacked adequate social support, and 21.3% had chronic depression symptoms [11], which are associated with worsened health outcomes [12].

Self-determination theory (SDT) provides a conceptual framework for considering how autonomy, competence, and relatedness may influence emotional well-being [13]. Relatedness highlights that the need for social connection [14,15] and lack of relatedness may give rise to emotional distress [14,16]. The symptoms can include depression, loneliness, anxiety, grief, and increased suicidal ideation [12,17,18]. While these constructs were not directly measured in this study, they provide context for understanding why peer support may be helpful for individuals experiencing emotional distress. These symptoms are widespread and have personal and population-wide consequences [9,12,19,20]. Together, depression, loneliness, despair, grief, work-related stress and anxiety may be shaped by individuals’ unmet social connection and disrupted relatedness over time [12,14,15].

This growing mental health epidemic highlights the urgent need for a more effective delivery of mental health care that enhances accessibility and scalability beyond the typical one-on-one mental health care model [21,22,23]. The need for scalable emotional support is further enhanced by shortages of qualified mental health professionals [24,25], and structural barriers to accessing typical mental health care (e.g., access to providers due to availability or physical barriers, high costs, and difficulty navigating insurance systems) result in an unmet need for mental health care services [26,27,28]. Evidence-based psychological therapies such as cognitive behavioral therapy (CBT), dialectical behavior therapy (DBT), among others, are gold-standard interventions [29,30,31,32,33,34,35]; however, limitations due to accessibility such as cost, as well as therapist availability and stigma, restrict their effectiveness [21,22,36] and highlight the need for accessible tools that can provide timely and continuous mental health support.

### 1.2. Digital Peer Support

Peer support is defined as the process in which individuals with similar experiences provide sub-clinical emotional, informational and social support to peers [30,37,38,39,40]. Some of the informational support can encompass psychoeducational (PE) resources, which offer structured and evidence-based information, and may help in strengthening coping skills [41]. Peer support is widely accepted as an effective and evidence-based approach that augments processing, engagement and emotional validation [38,42,43]. Notably, peer support has been shown to be effective in marginalized communities, including racial and ethnic minorities, veterans, low-socioeconomic-status families, and LGBTQ+ individuals [44,45,46,47].

Digital peer support (DPS) is a scalable tool that can offer mental health support. In addition to increased accessibility and availability, DPS also allows individuals’ emotional expression to be continuously assessed in a more organic manner. Understanding how indicators of emotional distress evolve among repeat users of peer support can aid in evaluating the potential for more accessible mental health care to reduce emotional distress over a longer period than previously studied.

DPS takes place over digital interfaces to deliver peer support through synchronous or asynchronous interactions, and it is typically delivered via web-based settings, mobile applications or text-based chat [37]. Digitally delivered mental health support is just as effective in reducing rates of depression, anxiety and other common mental health concerns [45,46,47,48,49,50,51,52]. By reducing stigma and removing financial barriers such as not necessitating insurance coverage [36,53,54], DPS may facilitate even more sustained engagement, especially for users who are unable or reluctant to access typical mental health services [55]. Additionally, moderation is essential to ensure user safety in such digital environments, especially when users experience crises that require fast and effective reactions [56,57,58,59,60]. In digital settings, PE resources can also be provided to facilitate more targeted support in response to the users’ concerns. When applied in real-time, they can reinforce behavior change, rather than just offering emotional validation [61,62].

DPS has established benefits on emotional well-being in single sessions. In a recent evaluation of a 24/7, synchronous, anonymous, and peer-moderated DPS service, negative sentiments declined by over 40% after a single session, while positive affect increased by 145% [57,58,63,64]. These results illustrate the immediate emotional benefits associated with participating in DPS. However, existing studies have not examined how these emotional benefits evolve, or how sociodemographic characteristics (e.g., gender, age, race or ethnicity) can affect users’ emotional trajectories with repeated DPS use.

Sociodemographic factors such as age, gender, race or ethnicity can influence how users engage with and benefit from digital mental health services [65,66]. Younger users show high rates of digital mental health literacy [67,68], while older adults may use digital tools less frequently despite a need for mental health support [69,70,71]. Gender differences are also reported [72,73,74,75]. Similarly, there are racial and ethnic variations in user engagement. Structural barriers, cultural stigma, views on health care systems, and accessibility concerns can all affect participation among populations, even in digital settings [76,77]. Understanding how sociodemographic factors affect engagement with mental health services like DPS can help improve equity and effectiveness of such tools. Furthermore, examining emotional outcomes associated with DPS use alongside demographic factors can help recognize how different populations benefit from digital mental health services and where there are differences in order to optimize support [78,79,80,81]. Emotional distress may vary over time and in response to global social stressors, such as recent geopolitical conflicts and the rising cost of living [82,83]. These conditions highlight the importance of examining individuals’ expressed sentiment over repeat sessions, rather than in a single-session analysis.

### 1.3. Gaps in Knowledge About Digital Peer Support

In addition to barriers in accessing formal mental health care, there are shortcomings related to how emotional distress is evaluated. Typical mental health care services rely on clinical questionnaires that assess individuals over extended periods and depend on individuals’ willingness to share concerns [84,85]. They are also vulnerable to recall bias, may not accurately reflect daily fluctuations in emotional expression, and have limited ecological validity [86,87]. Ecological momentary assessments (EMAs) overcome some temporal limitations but are not always possible to administer due to being resource-intensive and are limited to research settings [88,89]. These challenges underscore the need for scalable, synchronous approaches to support individuals.

When evaluating changes in emotional states, it is important to ensure that the examined construct is meaningfully present at baseline. Prior studies have commonly applied baseline thresholds when evaluating subsequent change, including studies of depression, anxiety, stress, and momentary negative affect [88,89,90,91]. Similar approaches have also been used in subclinical affective research, where changes in negative affect could be evaluated when sufficient negative affect was initially present [92,93]. Therefore, applying a baseline sentiment threshold can reduce floor effects and allow for the assessment of sentiment fluctuations. In this study, sentiment thresholding was used to identify users expressing a meaningful level of sentiment of interest at baseline.

Prior literature has examined sentiment trajectories in complementary and online support platforms, such as mental health forums, chronic or rare illness communities, and other tools [94,95,96]. Despite offering social support, most of these platforms are asynchronous and not moderated in real time, which creates a limitation of not offering immediate relief or ensuring user safety. Additionally, only a few studies have examined specific sentiment outcomes over multiple sessions [94,95,96].

Evidence for repeated peer support is only available for in-person settings and is limited [97,98,99]. Studies with follow-ups over multiple years indicated that peer support is associated with improved mental health outcomes, including reduced depression and anxiety and increased self-efficacy [97,98]. A study that evaluated college students over three years found that peer support bolstered self-efficacy and social adaptability [98]. Other observational studies indicated that peer support decreased psychological distress, as measured over multiple time points [97]. Peer support also has documented benefits in the single session, improving psychological well-being [100]. Online forums that include peer support have also demonstrated benefits in increasing self-efficacy and perceived social support [53].

As opposed to in-person peer support, studies of DPS are limited to shorter periods [37,48]. Studies examining DPS using randomized controlled trials and observational studies evaluated outcomes after a single session to a few months, but follow-up periods never exceeded a few months [50,101,102,103]. Some studies reported longer intervention durations up to multiple years; however, these studies relied on pre–post assessments and did not capture sentiment trajectories, and follow-ups may have occurred after DPS was long discontinued [37,48,104]. Despite these temporal limitations, many studies suggest DPS benefits are comparable to typical mental health care, reporting significant improvements in psychological well-being [37,48,63,101,105]. Additionally, the impact of PE resources on repeated DPS use is not yet established. Despite some studies reporting benefits of resource sharing in DPS [57,64,106], the impact of the frequency and timing of PE provision on sustained user sentiment is not documented.

While peer support has demonstrated longitudinal benefits, and DPS is a promising, more accessible alternative to in-person peer support, there is a lack of research examining how sentiments evolve over repeated sessions associated with real-world DPS engagement. Understanding these sentiment trajectories is critical to determining whether the benefits of DPS are sustained, and whether it can present scalable and equitable mental health support in the long term.

### 1.4. AI Tools in Digital Mental Health

As digital mental health services become increasingly common, maintaining user safety while optimizing responsiveness and personalization is critical. AI chatbots are increasingly used to seek digital mental health support by millions of users [107,108]. However, AI models commonly struggle with linguistic nuances, such as figurative language, sarcasm, slang or ambiguous statements that are often used in digital settings [109,110,111], and they are prone to validating pathological thinking patterns and are unable to provide support during crises [112,113,114]. Especially in high-stakes settings like health care, concerns about transparency of AI models highlight the importance of human supervision [115,116,117]. In the analyzed DPS service, AI-supported natural language processing (NLP) functioned as a real-time decision-support tool for human moderators. NLP provided topic-matching; supported human moderators by suggesting psychoeducational resources, flagging safety concerns for moderator review; and retrospectively classified sentiments. These functions were designed to support human moderators, rather than replacing them. Unlike autonomous AI chatbots, human moderators remained responsible for providing support, interpreting user needs, monitoring user safety, and initiating crisis escalation when needed.

Hybrid moderation approaches are used in some digital mental health settings; however, evidence of their effectiveness is limited to short-term studies. It is unclear whether repeated engagement with hybrid DPS services is associated with improvements in user sentiments. Examining emotional changes over time can clarify whether AI-assisted and human-moderated DPS is related to benefits beyond occasional DPS sessions.

### 1.5. Study Objectives

To address these limitations, this study examined sentiment patterns among repeat users of a real-time, synchronously moderated DPS service. The primary objective was to characterize changes in expressed negative sentiment across repeated DPS engagement. Secondary objectives were to descriptively characterize sociodemographic patterns and user engagement across conversation topics, including session frequency, return intervals, peer interactions, session duration, and PE resource provision. Together, these analyses were intended to characterize sentiment trajectories and patterns of repeated DPS engagement in a real-world setting.

## 2. Materials and Methods

### 2.1. Supportiv DPS Service Model

Supportiv is a real-time moderated, anonymous small group live chat service that operates synchronously and is available 24/7. It is a U.S.-based and paid service delivered through a browser interface that can be accessed via links from certain health plans or employer portals. Individuals choose to use the service voluntarily through an informed self-selection process. Before joining any live DPS chats, users must review and actively consent to the Terms of Use and Privacy Policy, including permission to use their anonymous data, which is inherently anonymous from the outset, not anonymized post-collection, for research and service improvement purposes. No personally identifiable information (PII), protected health information (PHI), or HIPAA-protected health information is required. Any potential personal identifiers are removed, and data is stored in a de-identified format. All data handling complies with the General Data Protection Regulation (GDPR) and California Consumer Privacy Act (CCPA) laws. Access to data is restricted to authorized personnel only.

Engagement begins when users respond to the prompt “What’s your struggle?” AI-driven natural language processing (NLP) matches users with peers facing similar challenges, forming small groups of up to five anonymous participants. Each group is facilitated live by a hybrid solution in which an AI model supports a sub-clinically trained human moderator. Moderators facilitate discussion flow, enforce chat rules to maintain a psychologically safe environment, share targeted and useful resources that are suggested by a patented AI-matching technology, and remove users for inappropriate behavior. Synchronous moderation is performed with interactive oversight during live sessions, rather than asynchronously or post hoc. Moderators also facilitate collaborative problem solving, and in cases of crisis, promptly route users via links to professional crisis services to provide comprehensive support.

### 2.2. Study Design and Participant Enrolment

This retrospective observational study analyzed anonymous 13,686 live chat sessions from 8084 distinct users stored in Supportiv’s secure database between January 2023 and December 2024. The main goal was to track repeat users and assess whether initially high negative sentiment decreased over successive sessions.

Because the service is anonymous and does not collect persistent personally identifying information, repeat-user linkage relied on non-sensitive device/browser characteristics rather than verified individual identifiers (such as browser and screen resolution). Only minimal metadata, including service-return patterns and optional user-reported demographic information, were collected. Geographic context was determined using truncated IP addresses to allow city-level analyses without identifying individuals. All data was stored in a de-identified format.

Repeat user linkage accuracy could not be confirmed against a direct identifier; therefore, some returning users may not be linked, or, less commonly, separate users with similar device characteristics may be linked.

### 2.3. Data Acquisition, Preprocessing, and Topic Annotation

GPT-4o-mini was used for both topic annotation and sentiment scoring based on expressed language. The chat messages were classified into five overarching topics (grief, loneliness, depression, suicidality, and work-related). These topics were determined based on the most frequently discussed conversation topics in chat transcripts. The model was trained with expert-curated text examples according to predefined clinical criteria. Since a participant could address several issues in a single conversation, the model was allowed to assign multiple topics per session. Because sessions could be assigned to more than one topic, resulting topic-specific groups were not mutually exclusive and could contain overlapping observations. Thus, returning users received a separate topic list for each visit, enabling us to track how sentiment trajectories change when the same concern reappears across chats. These topic labels were used to determine which conversations were included in the topic and sentiment analyses. For example, sessions classified into the depression topic were included in the depression sentiment analyses, and sessions classified into suicidality-related topics were included in the despair sentiment analyses. To reduce the model’s errors in classifying topics, outputs were constrained to a predefined topic ontology, and classifications were manually reviewed. There remains a risk of excluding possible conversations, misclassifying topics, or analyzing sentiment based on inaccurate topic classifications. A formal inter-rater agreement was not calculated for topic classification.

The conversation-level records represented a single peer support chat session and included metadata, such as system-assigned anonymous user ID, timestamps, session duration, message counts, topic labels, and optional self-reported demographics, along with emotion scores previously calculated by validated emotion analyzers. These fields enable chronological ordering of chat sessions and analysis of patterns by topic and subgroup. Session start times were converted to Coordinated Universal Time (UTC) for chronological sorting. For every session, only the messages written by the primary participant were observed.

### 2.4. Sentiment Scoring

Few-shot prompts included expert-curated examples and predefined topic and sentiment definitions. We included every chat that contained at least one of the predefined topics in our analyses (suicidality, work-related, grief, loneliness, and depression). For each session, the ordered sequence of messages, together with user metadata, was converted into a context object and processed with the sentiment-analysis pipeline from our earlier work [57,58,63]. The sentiments that were analyzed were suicidality, work-related stress, grief, loneliness, and depression. Suicidality was quantified as despair for sentiment trajectory analysis.

The sentiment analysis pipeline uses a few-shot prompt to give GPT-4o-mini a small set of labeled examples, guiding its predictions. Prior validation of this approach used a few-shot framework with 10 labeled examples, including five positive and five negative examples, and the sentiment-scoring pipeline instructed the model to estimate sentiment intensity [57]. These examples were peer support chat messages that were manually curated by clinical psychology experts. Each eligible message written by the primary user then received a time-stamped intensity score for the target sentiment emotion of interest. Messages lacking measurable sentiment were excluded from analyses. Scores range from 1 (low) through 5 (moderate) to 10 (high), providing enough granularity to capture subtle shifts while remaining easy to interpret. This scoring method, including sentiment definitions and intensity scoring, has been validated against human raters and showed consistency across multiple sentiments [57]. Prior examinations have demonstrated that this method performs comparably to typical sentiment classification instruments [118].

The sentiment scores were inferred from user-expressed language and were not intended to diagnose psychiatric symptoms, measure symptom severity, or substitute for validated psychometric instruments such as the PHQ-9, GAD-7, or PSS-10. Changes in sentiment scores should be interpreted as changes in expressed affective language in-the-moment, rather than clinical outcomes.

The sentiment scoring approach was previously validated against expert human ratings, as described in prior work [57]. In that validation, more than 2500 model outputs, representing 25% of outputs, were manually reviewed, with additional review of high-uncertainty messages to help experts identify and iteratively fine-tune potential errors in nuanced language, such as metaphors and sarcasm. This approach allowed identifying misclassified messages. Full methodological details are provided in the original study [57]. This study applied this previously validated pipeline to the longitudinal dataset, and a separate formal human-versus-model agreement analysis was not performed.

### 2.5. User Inclusion Criteria

To ensure that the chat sessions included measurable sentiment, sessions were included in analysis based on the availability of sentiment scores. A session was included in the analysis only if it contained at least three messages with assigned sentiment scores. Chat messages that lacked measurable content were not scored and were therefore excluded from analyses.

For each user, sessions were sorted chronologically, yielding 13,686 chat records from 8084 distinct users. A user was included in analyses if they had a minimum of two sessions that met the sentiment scoring threshold (above or equal to 5), and each session contained at least three sentiment-scored data points. The ≥5 threshold was selected to restrict the longitudinal analyses to user conversations with sufficiently present target negative sentiment to allow observable change. On the 1–10 language-based expressed sentiment scale, a score of 5 represents moderate expression of the sentiment of interest. Restricting analyses to observations with an elevated baseline sentiment is consistent with prior studies that evaluated changes in depression, anxiety, stress, and momentary negative affect, which have also applied a similar baseline threshold before assessing subsequent changes [88,89,90,91,92,93]. This approach reduces floor effects and avoids estimating decreases in conversations in which studied sentiments were minimal at baseline. This threshold is an analytic criterion and not intended to represent clinical diagnoses or symptoms. Additionally, requiring at least two sessions and at least three scored messages per session provided repeated observations to characterize user sentiment trajectories over repeated use. The sentiment scoring threshold identified the user’s first high-intensity encounter on a given topic, recognizing that conversations often begin with casual greetings. All subsequent sessions in which the same topic re-emerged were then included in the analysis. A total of 596 unique users met these inclusion criteria, and 4099 sessions were analyzed for this study. The inclusion criterion selected users with relatively high initial sentiment; therefore, trajectory analyses may be prone to regression to the mean, natural emotion settling over time, or how moderators guided the conversations.

### 2.6. Sentiment Analysis

Users who met the inclusion criteria were analyzed individually. For each user, sentiment scores were linked across sessions, with elapsed time (minutes) on the x-axis and sentiment intensity on the y-axis. To quantify within-user changes in sentiment, simple ordinary least squares (OLS) linear regression was calculated to estimate the slope and statistical significance of each user’s sentiment trajectory, quantifying how their affect evolved across repeated discussions of the same struggle topic. Because the analysis centered on negative sentiments, improvement was represented as a negative slope, indicating declining sentiment intensity over time. Statistical significance of the slope was evaluated using a two-sided Student’s *t*-test, which tested the null hypothesis that the slope is equal to zero, with no change in sentiment intensity. These per-user OLS analyses were used to descriptively characterize individual sentiment trajectories rather than estimate population-level effects, and they do not account for the clustering of messages within sessions.

To summarize the distribution of per-user OLS trajectories within each topic–emotion pair, users were categorized according to the direction of their sentiment-score-fitted slope and whether the individual OLS slope reached the *p* ≤ 0.05 threshold. The sentiments that were matched to struggle topics were depression, grief, loneliness, despair, and work-related stress. Combining these parameters yielded four possible outcome categories for every topic-sentiment pair:

1. Negative slope, significant;
2. Negative slope, non-significant;
3. Positive slope, significant;
4. Positive slope, non-significant.

A significant negative slope (category 1) represented declining negative sentiment across accumulated conversation time for that individual, whereas a positive slope (category 3) represented increasing sentiment. Statistical significance refers to individual OLS slopes. To summarize these trajectory patterns, we computed two summary ratios:

- Significance-adjusted ratio: users in category 1 divided by all users whose slopes were significant (categories 1 + 3).
- Direction-adjusted ratio: users in category 1 divided by all users with negative slopes (categories 1 + 2).

These ratios were used to descriptively summarize the distribution of OLS trajectory directions across users and were not estimates of population-level change.

### 2.7. Sensitivity Analyses

To assess the strength of the longitudinal sentiment trajectory analyses, sensitivity analyses were carried out. These sensitivity analyses tested how the results varied when (1) only users with three or more sessions were included in analyses rather than two, (2) the operationalized time was defined as the retained session number rather than accumulated message time, and (3) the baseline sentiment threshold was changed as ≥4 or ≥6, rather than ≥5. For each sensitivity analysis, the same per-user trajectory analysis was used, and the significance- and direction-adjusted ratios with 95% confidence intervals were calculated for each topic–emotion pair.

### 2.8. Resource Provision

To reduce the influence of extremely high-resource sessions on mean resource-count estimates, resource-count analyses applied topic-wise Tukey outlier removal. Within each topic, sessions with resource counts greater than Q3 + 1.5 × IQR were excluded before calculating mean resources per session and conducting Mann–Whitney U comparisons against the aggregate distribution. This approach was used since resource counts were highly right-skewed, with a small number of sessions containing unusually large numbers of shared resources that could disproportionately affect topic-level means. This approach yielded 588 users analyzed for resource distribution.

### 2.9. Statistical Evaluation

Statistical tests addressed two objectives: (1) comparing pairs of data groups for a given feature and (2) determining whether an observed trend was significant. Because the data were not normally distributed, non-parametric methods were applied.

For comparisons between independent groups, the Mann–Whitney U test was used. For within-user repeated-use analyses, statistical significance of sentiment trajectories was determined using a two-sided Student’s t-test on the slope of the linear regression line, testing whether the slope differed from zero.

Demographic analyses were exploratory since users provided their demographic information voluntarily. Differences in topic patterns based on demographics were not evidence of varying effectiveness. Since some subgroups have small group sizes, results were used to describe observational findings. Sensitivity analyses were not applied for demographic subgroup analyses.

Topic-specific engagement and session-characteristic summaries were descriptive. The overall ‘ALL’ group was included, and comparisons between individual topics and the ‘ALL’ aggregate were not performed.

### 2.10. Ethical Approval

This study was conducted retrospectively on an anonymous dataset and was deemed exempt from human ethics research review by the Pearl Institutional Review Board under protocol no: IRBSUPP0021.

## 3. Results

### 3.1. User Engagement and Demographics

#### 3.1.1. User Distribution

A total of 13,686 anonymous peer support live chat sessions from 8084 unique users were present. A total of 4099 chat sessions from 596 unique repeat users met the inclusion criteria and were analyzed. The conversations took place from January 2023 to December 2024. Out of 596 users, 306 self-reported their age (51.3%). The median age of users was 41.0 (μ = 50.2, SD = 28.8). Out of users who met the inclusion criteria, only 38.9% (*n* = 232) reported their gender, and out of these users, 71.6% identified as female (*n* = 166) and 28.5% as male *(n* = 66). A total of 41.4% (*n* = 247) of all users disclosed their race/ethnicity background. Of these users, those identifying as White comprised the majority (*n* = 142, 57.5%). Hispanic or Latino users constituted 13.0% of users (*n* = 32), Black or African American users made up 12.6% of all users (*n* = 31), and Asian users accounted for 9.3% (*n* = 23). The least frequently represented ethnicities were American Indian or Alaska Native (4.5%, *n* = 11), Native Hawaiian or Pacific Islander (2.4%, *n* = 6), and Middle Eastern or North African (0.9%, *n* = 2).

#### 3.1.2. User Engagement Patterns by Topic

User engagement varied significantly by topic (Table 1). User conversations that included more than one sentiment were included in multiple analyses. Depression (*n* = 317) and work-related (*n* = 303) concerns attracted the largest cohorts, followed by grief (*n* = 198) and loneliness (*n* = 174). Suicidality was discussed by substantially fewer users (*n* = 34), compared to the other categories and well below the overall topic average (*n* = 205). On average, users participated in 4.00 sessions (σ = 4.43) across different topics. Three topics clustered tightly around this mean: grief (μ = 4.08, σ = 4.17, *p* = 0.750), loneliness (μ = 4.18, σ = 4.34, *p* = 0.074), and depression (μ = 4.37, σ = 5.31, *p* = 0.345) showed no significant differences from the overall distribution. Users discussing suicidality participated least often (μ = 2.88, σ = 1.81, *p* = 0.102), yet this difference was within the overall variance and was not statistically significant, possibly due to the small sample size. The only topic with a significantly reduced session count was the work-related category, in which users completed significantly fewer sessions (μ = 3.57, σ = 3.75; *p* = 0.041).

As opposed to the individual-level engagement patterns in Table 1, differences in population-level engagement between session volume and user count emerged by comparing each topic’s proportion of total sessions (Supplementary Table S1). Depression-related concerns dominated session volume (33.8%, *n* = 1384) and accounted for a slightly smaller share of the user base (30.9%, *n* = 317), indicating repeated engagement among users who discussed depression. In contrast, work-related conversations claimed a slightly larger proportion of users (29.5%, *n* = 303) than sessions (26.4%, *n* = 1083), suggesting those users visit less frequently. Grief, loneliness, and despair showed nearly one-to-one parity between sessions and users, implying comparable rates of user engagement across these topics. Sessions focusing on grief had a session volume of 19.7% (*n* = 807) with 19.3% user share (*n* = 198), and loneliness had a session volume of 17.7% (*n* = 727) with 17.0% user share (*n* = 174). Sessions were least frequently focused on suicidality, with a session volume of 2.4% (*n* = 98) and 3.3% user share (*n* = 34).

#### 3.1.3. Gender Distribution

A key goal of this study was to examine the gender distribution among returning users. Only users who met the inclusion criteria and voluntarily reported their gender were analyzed (38.9%, *n* = 232). The overall male-to-female ratio was 0.40, since there were more female users. Depression (0.39), grief (0.38), and work-related (0.38) discussions were aligned with the average ratio, with slightly more women discussing depression, grief and work-related topics. In contrast, loneliness showed a slightly higher ratio (0.43), while suicidality was markedly lower (0.31). Among users who voluntarily reported their gender, men used DPS services repeatedly to discuss loneliness, whereas women engaged more in repeated conversations about suicidality. Since all ratios were below 0.50, returning users were predominantly female, consistent with prior findings that women are more likely to engage in digital mental health services [114]. However, gender reporting was voluntary and incomplete, and topic-specific user counts were small, especially with regard to suicidality. These findings should be interpreted as descriptive rather than absolute gender-based differences.

#### 3.1.4. Race and Ethnicity Distribution

Race and ethnicity distributions were descriptively examined among users who voluntarily reported their demographic information (41.4%, *n* = 247). Among these users, there were some differences in user engagement with DPS overall, as well as across topics (Supplementary Table S2). However, these differences should be interpreted with caution and should not be evidence of definitive racial or ethnic differences, as some subgroups were very small in size, and demographics were voluntarily reported. The distribution of White users varied across topics, with lowest engagement for grief (51.6%, *n* = 49) and highest for suicidality and loneliness (61.1%, *n* = 11 and 59.3%, *n* = 54, respectively) topics. Black or African American users represented 16.7% of users for suicidality-related discussions (*n* = 3) and 14.6% of users in depression-related discussions (*n* = 20). Hispanic or Latino users represented 14.7% of users in grief-related discussions (*n* = 14), while Asian users represented 11.1% of users in suicidality-related discussions (*n* = 2) and 11.0% of users in loneliness-related discussions (*n* = 10). Other racial and ethnic subgroups had very small sizes for discussion topics and were reported descriptively without further interpretation. Overall, these data provide a descriptive overview of the demographic breakdown of users who voluntarily reported their race or ethnicity, but the low user numbers for certain discussion topics limit subgroup comparisons.

### 3.2. Sentiment Trajectories Across Repeated DPS Use

#### 3.2.1. User-Level OLS Sentiment Trajectories

To describe individual sentiment patterns across repeated engagement, user sentiment scores from different sessions were plotted, and a trajectory line was fitted for each user. Figure 1 shows a returning user who participated in 13 chat sessions focused on depression. The x-axis plots the cumulative session time in minutes, while the y-axis shows the depression sentiment score assigned to each of the user’s messages (individual-colored dots). For this example repeat user, 13 sessions and 315 messages were included in sentiment analyses. The fitted regression line had a slope of −0.00386 depression score points per accumulated minute (95% Cl: −0.00501 to −0.00270), corresponding to an estimated decrease of 2.73 points on the 1–10 sentiment scale. There was a modest variance in depression scores (R^2^ = 0.121; r = −0.348), indicating a measurable but varying downward trend.

To describe user-level sentiment trajectories across topics, we analyzed per-user OLS trajectories for five sentiments: depression, grief, loneliness, despair, and work-related stress (Table 2). For each sentiment, users were categorized based on the slope direction and statistical significance of the fitted trajectories. Outcomes were summarized as the significance-adjusted ratio, defined as the ratio of users with significant negative slopes relative to all users with statistically significant slopes. These measures were used to describe the direction and statistical significance of user-level sentiment trajectories.

Across all topic–sentiment pairs, significance-adjusted ratios exceeded 70% (*n* = 4099), and most exceeded 85% (*n* = 2289). Suicidality|despair led, with 90.9% of trajectories that were significantly trending downward (*n* = 34), followed by depression|depression (86.1%, *n* = 317), grief|grief (85.7%, *n* = 198) and work-related|stress (82.4%, *n* = 303). Despite a lower improvement when compared to other pairs, loneliness|loneliness still showed considerable change in the long term (72.7%, *n* = 174). It should be noted that suicidality|despair had a small sample size and wide confidence interval due to the relatively infrequent nature of the conversations. These findings descriptively summarize that users demonstrated declining negative sentiment across accumulated conversation time.

When focusing on significant downward trajectories versus all negative slopes, an average of 45.5% of all analyzed trajectories within each topic–emotion pair shifted in a significant and downward direction (Table 2). Grief|grief showed the highest proportion of significant downward individual trajectories (55.3%, *n* = 198), followed by depression|depression (44.7%, *n* = 317), loneliness|loneliness (44.0%, *n* = 174), and work-related|stress (43.3%, *n* = 303). Suicidality|despair demonstrated a slightly lower but still substantial portion of significant improvement (40.0%, *n* = 34). These findings suggest that a meaningful subgroup of users showed significant downward individual trajectories in expressed negative sentiment, especially in grief, depression, and suicidality contexts.

#### 3.2.2. Sensitivity Analyses

Sensitivity analyses mainly supported the direction of the user-level sentiment trajectories, but the effect sizes of the findings depended on the analysis parameters. When users with only two sessions were excluded, most of the significant trajectories remained in the negative direction. The significance-adjusted ratios ranged from 66.7% to 79.7%, and the direction-adjusted ratios ranged from 28.6% to 54.4% (Supplementary Table S3). However, the suicidality|despair topic–emotion pair had a smaller sample size of 11 users and 52 conversations.

When the session number was used instead of the accumulated message time for analyses, the findings were consistent with the main results, but some had smaller effect sizes. Trajectories that were significant were mostly negative, with significance-adjusted ratios ranging from 52.5% to 74.4% and direction-adjusted ratios ranging from 26.5% to 45.3%. The strongest topic–emotion pairs were depression|depression and grief|grief, whereas work-related stress and loneliness sentiments had weaker ratios.

When the baseline sentiment was changed from ≥5 to ≥4 or ≥6, the number of users and sessions changed as expected, but the direction of the sentiment trajectories remained consistent with the original findings. These sensitivity analyses showed that the predominance of negative slopes in the descriptive per-user OLS trajectories was generally maintained, although the magnitude of summary ratios depended on analytical parameters. The detailed sensitivity analysis results are included in the Supplementary Table S3.

### 3.3. User Engagement and Behavior

#### 3.3.1. Peer Interactions

Across all topics, users interacted with a small number of peers per chat session (μ = 1.43, σ = 2.15) (Supplementary Table S4). Topic-specific averages were similar, ranging from 1.3 to 2.1 peer interactions, with comparable standard deviations (σ = 2.0–3.3). Although suicidality-related sessions showed higher amounts of peer interactions (μ = 2.09, σ = 3.31) (*n* = 98), they did not reach statistical significance due to the smaller sample size and higher variation in interaction patterns (*p* = 0.067). Overall, these findings suggest that most users interact with only one or two peers in a session, regardless of the topic of the discussion.

#### 3.3.2. Average Time Between Sessions

As shown in Table 3, the average time between sessions differed across discussion topics. On average, users waited 10.4 days before seeking DPS again (σ = 28.6). Users discussing depression (μ = 9.6 days, *n* = 317 users, *n* = 1384 sessions) returned significantly sooner than average users (*p* = 0.012). Users discussing grief topics (μ = 9.4 days, *n* = 198 users, *n* = 807 sessions) returned slightly sooner than average, while users discussing work-related topics returned around the average rate (μ = 10.8 days, *n* = 303 users, *n* = 1083 sessions). However, neither of these differences was statistically significant from the average (*p* = 0.550 and 0.651, respectively), suggesting similar return patterns across users discussing grief and work-related topics. Users discussing loneliness delayed following up longer between sessions (μ = 11.8 d, *n* = 174 users, *n* = 727 sessions; *p* = 0.034), and those discussing suicidality waited the longest, with intervals nearly twice the overall average (μ = 18.6 d, *n =* 34 users, *n* = 98 sessions; *p* =1.56 × 10^−6^). In general, while most topics are revisited on around a ten-day cycle, users discussing loneliness and suicidality exhibit markedly extended intervals.

### 3.4. Session Characteristics

#### 3.4.1. Session Duration

On average across all topics, chat sessions lasted 51.28 min (σ = 46.26) (Supplementary Table S5). Sessions discussing depression (μ = 49.43, σ = 42.35, *n* = 1384), grief (μ = 50.76, σ = 45.44, *n* = 807), work-related (μ = 51.5, σ = 49.23, *n* = 1083), and loneliness (μ = 53.65, σ = 44.97, *n* = 727) were similar in length and did not have significant differences (*p* = 0.336, 0.547, 0.655, 0.052, respectively). In contrast, sessions focused on suicidality were approximately 20% longer and more variable (μ = 61.85, σ = 72.9, *n* = 98; *p* = 0.049).

#### 3.4.2. Resource Distribution

In order to determine the average number of PE resources that were shared for each typical chat, user conversations with outlier amounts of resources were excluded from analyses, yielding 588 total analyzed users. On average, users received 9.19 PE resources per session (Table 4). These PE resources included a variety of formats and materials to promote behavior change, including research-based information, peer narratives, and tools for skill-building like coping strategies and mindfulness exercises.

User conversations that focused on work-related, grief, and depression topics were near this average, with 9.48, 8.29, and 8.59 resources, respectively. In contrast, sessions discussing loneliness and suicidality included more shared resources, with 10.44 and 12.44 resources on average. This pattern indicates that moderators provided more psychoeducational resources, coping tools, or crisis links during more emotionally intense conversations that may have had increased risk to user safety. The greater variation in suicidality sessions may also show that resource sharing was tailored to user needs; some participants may have required only a few, while others may have benefitted from more resources.

## 4. Discussion

### 4.1. Principal Findings

Prior studies of DPS across randomized as well as observational designs have shown that DPS is associated with improved sentiment, including reduced loneliness, depression, and anxiety [57,58,101,103,119]. This study examined sentiment patterns across repeated DPS engagement to characterize sentiment trajectories across accumulated conversation time. Results showed that negative slopes were prevalent among statistically significant trajectories. The strongest positive associations over repeated sessions were observed in high distress-related sentiments like despair and depression, while their loneliness scores reflected significant yet moderate changes, possibly because social isolation requires additional time to resolve. Sensitivity analyses supported the direction of sentiment trajectories, even when the sentiment baseline thresholds were changed, and only users with three or more sessions were analyzed. However, when the number of sessions was used as a model variable, the trajectory magnitudes were altered.

The descriptive downward trajectories observed for some users may reflect several processes; however, the present study cannot determine their causes. Prior longitudinal research on in-person peer support spanning up to three years indicated that repeated peer interactions improved mental health through promoting social connectedness, empathy, and validation, which all in turn enhance users’ emotional regulation and self-efficacy over time. For example, a study spanning three years in college students found that in-person peer support was linked to increased self-efficacy, leading to better mental health outcomes [98]. Two other studies with adolescent cohorts aged 11 to 17 years demonstrated that peer support was associated with reduced depression and anxiety, though this relationship may be bidirectional [97,99]. In this context, the benefits of peer support can be further reinforced by motivating behavior change in participants through PE resources [41,50,61]. In agreement with social cognitive theory and the transtheoretical model of change [120,121], moderators can provide resources to further guide and provide emotional validation in order to promote therapeutic peer engagement.

These findings should also be considered from the perspective of self-determination theory (SDT) [15]. DPS interactions may promote relatedness by increasing social connections and validation by peers. This is especially important for users experiencing loneliness. PE provision may strengthen users’ competence by offering helpful resources and perspective. Since DPS is user-initiated, it may support feelings of autonomy, allowing emotional support at the pace users need. These constructs may be relevant to how users experience repeated DPS use, but the present study did not directly assess these mechanisms.

Engagement patterns varied by topic and revealed important trends in DPS users. Users sought support sooner after discussing depression (9.6 days as opposed to an average of 10.4 days) (Table 3), which indicated a need for sustained support. On the other hand, users discussing loneliness and suicidality returned to DPS after longer periods between sessions, possibly due to increased processing time or emotional load (11.8 days and 18.6 days, respectively). Sessions focusing on suicidality were longer and had a higher variation (61.85 min, σ = 72.90), possibly due to the smaller sample size and increased number of outliers (Supplementary Table S5), and involved more resource distribution (12.44 resources, σ = 12.30) (Table 4), all of which point to moderators’ safety-aligned support for users with high acuity. Since engagement patterns reflect differences in emotional processing across topics, understanding these differences is important for optimizing moderator intervention strategies and timing.

Sociodemographic analyses showed that the median age was 41, and most DPS users were female, in agreement with prior research that women are more likely to seek digital mental health services [122,123]. While White users were the majority (57.5%), Black and American Indian/Alaska Native users were more represented in sessions focusing on suicidality (16.7% and 5.6%, respectively, in suicidality-related conversations) (Supplementary Table S2). Users’ demographic distribution suggests that the service was used by individuals from multiple racial and ethnic groups; however, self-reported demographic data is incomplete, and small subgroup sizes do not allow for conclusions regarding equitable access across all demographics. Future studies with more complete and verified demographic information and subgroup analyses could determine whether user engagement and sentiment outcomes statistically differ by gender, race/ethnicity, or other factors.

These findings highlight DPS services’ potential to provide a scalable, accessible, and anonymous complement to formal mental health care. DPS may provide accessible emotional support through repeated engagement. However, this study did not directly assess factors such as resilience, psychological growth, or clinical outcomes. Notably, user engagement was dependent on discussion topics, indicating that users sought DPS depending on their emotional needs. Session characteristics also showed user engagement depending on user needs. Conversations involving suicidality were longer, more variable and included more shared resources. These patterns indicate that moderator activity and resource provision varied across conversation topics.

This study should also be considered in relation to asynchronous DPS tools, including online health communities, mental health forums, and social media platforms. Asynchronous settings can also offer scalable and flexible access to peer support and similarly may provide support to individuals outside typical mental health care services in clinical settings [124,125,126]. However, it is important to note that asynchronous peer support services can have limitations in moderation, information quality, and maintaining user safety. In these settings, delayed responses and exposure to distressing, inaccurate or inappropriate content may reduce their benefits [53,127,128]. Contrastingly, the DPS model in our study was moderated synchronously and allowed users to engage with their peers in real time with human moderator oversight. A key advantage of synchronous moderation over asynchronous tools is the ability to provide real-time human oversight, respond immediately to safety concerns, direct users to crisis resources when needed, and provide appropriate psychoeducational resources at times of need. These benefits may be especially important for users who are experiencing loneliness, despair, or crisis-related concerns. However, this study did not compare synchronous DPS with other services.

Importantly, this study addresses a critical gap in knowledge in the DPS literature. Although other studies report longer follow-up periods, they are heterogeneous in design, collect pre–post assessments without continuous sentiment trajectories, and report follow-ups that may occur after interventions have been completed for a while [48,104]. Examining real-time, real-world engagement with DPS over multiple years presents novel evidence on associated sentiment outcomes, building on the prior literature. However, this study did not compare outcomes between users’ sentiment outcomes from a single session versus repeated session DPS use, nor can it infer causality between long-term DPS use and sentiment improvements due to its retrospective and observational design.

These results should also be interpreted within the context of the study period in 2023 to 2024, which was characterized by political conflicts and economic instability. These large-scale stressors can significantly increase emotional distress and affect individuals’ help-seeking behaviors, especially with mental health support. DPS may be an effective tool for providing around-the-clock emotional support, even in the presence of persistent global stressors.

### 4.2. Limitations and Future Directions

Despite establishing novel associations with repeated DPS, some limitations should be taken into account when interpreting these results. First, the study is retrospective and observational, and therefore the sentiment changes cannot be causally and solely attributed to DPS use. In addition, the inclusion criterion selected users with relatively higher baseline negative sentiment, which may increase the likelihood of subsequent declines through regression to the mean independent of DPS engagement. Second, per-user sentiment trajectories combine observations across multiple sessions and may reflect within- and between-session changes. Third, users self-selected into the service and into repeated use, creating a possible selection bias because repeat users may differ from other users in their motivation, needs, or openness. Finally, sentiment scores were derived from AI-based analysis of expressed language; therefore, changes should be interpreted as changes in expressed affective language and not clinical outcomes. Future analyses using mixed-effects models could consider the hierarchical structure of user messages to distinguish between within- and between-session changes. Since the dataset was a naturalistic conversational DPS dataset with heterogeneous users, differing intervals, and number of sessions, a generalizing population-level approach may not be able to capture the meaningful variability among users.

OLS analyses provide a granular approach to user-level sentiment changes, but they do not take the dependent nature of the same users’ repeated sessions into account. There are other shortcomings with using the same trajectory analysis for every user, since users have different numbers of sessions and time intervals between sessions. The per-user OLS analysis characterized sentiment trajectories across accumulated conversation time but did not model messages nested within users. Because messages from multiple sessions contributed to a single trajectory, these slopes may capture both within-session and between-session sentiment changes.

Since users are self-selected, as they autonomously decide to use the DPS service, this may limit generalizability to a wider population, since some individuals may be less likely to seek out mental health support, let alone on digital platforms, or show openness to share their emotional struggles. Users who sought DPS and returned to the service for repeated support may differ from the rest of the population in terms of motivation or readiness for behavioral change and openness to peer support, creating a potential user attrition bias. Furthermore, demographic information, including age, race, and gender, is self-reported and only available for a subset of users, which introduces the possibility of a reporting bias. Incomplete or inaccurate disclosure of self-reported demographic data cannot be confirmed due to the anonymous nature of data collection. Users who disclosed personal information may also differ from other users with regard to their openness, motivations, privacy preferences, and trust in mental health care or digital tools. Additionally, some subgroups contained a small number of users, especially when users with suicidality concerns were analyzed. These smaller samples may limit the accuracy of conclusions regarding demographic representation and must be considered as descriptive. The findings may not be generalized to all DPS users, including non-repeat users.

Notably, Supportiv, like other DPS services, does not assign clinical diagnoses; therefore, the interventions reflect sub-clinical categorization. Additionally, the sentiment scores are inferred from users’ language-based sentiment, rather than an assessment by clinical providers. Although this method allows for an organic capture of sentiment states, it may be prone to selection bias. Additionally, repeat users were identified using digital fingerprinting by device type and minimal location data, rather than sensitive identifiers, to maintain user anonymity. Due to this, it is possible that there was a misclassification of repeat users and that certain users who had been missed but returned were categorized as novel users. This limitation may affect repeat users’ analyses and should be considered when interpreting results. The anonymous and digital nature of this DPS service introduces other confounding variables, such as differences in digital literacy, employment, socioeconomic status, health care accessibility through insurance, employer, or independent sources and familial or friend social support. These variables may affect users’ engagement, as well as the sentiment outcomes measured over repeated sessions. Since these variables were not accounted for, they may affect the results.

Another challenge in interpreting conclusive results is the variability that is caused by the repeated tracking of user engagement. Over time, differences in user engagement patterns can influence both the usefulness of coping behaviors and the levels of benefits users receive from using the DPS service. Additional confounding variables, such as geopolitical events, economic conditions, individuals’ major life events, or receiving mental health care elsewhere, may also affect engagement and sentiment trajectories over time, complicating attributing outcomes to DPS use. Another variable is the depth of peer interactions. While this study examined the duration of sessions and number of peers that users interacted with, it did not capture the depth of peer interactions. Therefore, it is not possible to deduce whether users engaged with the same peers and held deeper conversations, and the findings do not differentiate between sustained and fleeting peer interactions.

The nature of the user inclusion criteria that required relatively higher baseline negative sentiment scores (≥5) may have increased the likelihood of observing sentiment decline due to regression to the mean. By selecting users during periods of relatively high emotional distress, the following scores may shift towards more typical levels over time, not fully attributable to DPS use alone. Without a randomized and controlled study design, it is not possible to causally attribute repeated DPS engagement to sentiment improvements, as outcomes may be influenced by other variables like regression to the mean, natural settling, external support, or other factors. Controlled studies could assess how different baseline sentiments are affected by repeated DPS engagement, while controlling for confounding variables.

Future studies could address some of these limitations to more precisely draw conclusions about the benefits of DPS use. While there is prior evidence indicating that DPS is associated with improved sentiment following a single session, this study did not directly evaluate the differences in sentiment outcomes between single-session and repeated DPS use. Future studies employing longitudinal randomized controlled designs with standardized follow-up intervals can better examine the effects of repeated DPS use without confounding variables and isolate the added benefit of repeated DPS use over time. Also examining dose–response relationships between session frequency, duration and resource distribution could further enhance our understanding of DPS benefits, as well as how DPS contributes to improved well-being. Future research should also examine the nature of peer interactions, including whether repeated interactions with the same peers, as opposed to different peers of varying numbers (one-to-one, or small groups of two, or larger groups of three or more), contribute to sustained sentiment outcomes. Additionally, although this study examined sociodemographic distributions of DPS users, future research should focus on interaction analyses between gender, age, and race or ethnicity to determine whether sentiment outcomes differ and assess equity in effectiveness.

## 5. Conclusions

This two-year retrospective observational study characterized sentiment trajectories among repeat users of a synchronously moderated DPS service. Across depression, loneliness, grief, despair, and work-related stress, some users demonstrated downward trajectories in expressed negative sentiment across accumulated conversation time, and negative slopes predominated among statistically significant individual trajectories. Most of the earlier studies investigated the short-term effects of DPS use, whereas this study examined how expressed negative sentiments changed with repeat DPS, including depression, loneliness, stress, grief, despair, and work-related stress.

Topic-specific differences in session duration, resource provision, and return intervals further characterize how repeat users engaged with the service. However, the observational design, absence of a control group, selection of users with relatively high initial sentiment scores, and inability of the analyses to distinguish within-session from between-session changes limit interpretation of the findings, and they do not establish causality.

Prospective longitudinal and randomized studies using standardized follow-up intervals and distinguishing between within- and between-session changes are necessary to determine whether repeated DPS use results in sustained sentiment improvements. DPS remains an accessible and scalable form of subclinical emotional support, but its longer-term effects require further investigation.

## Figures and Tables

**Figure 1:**
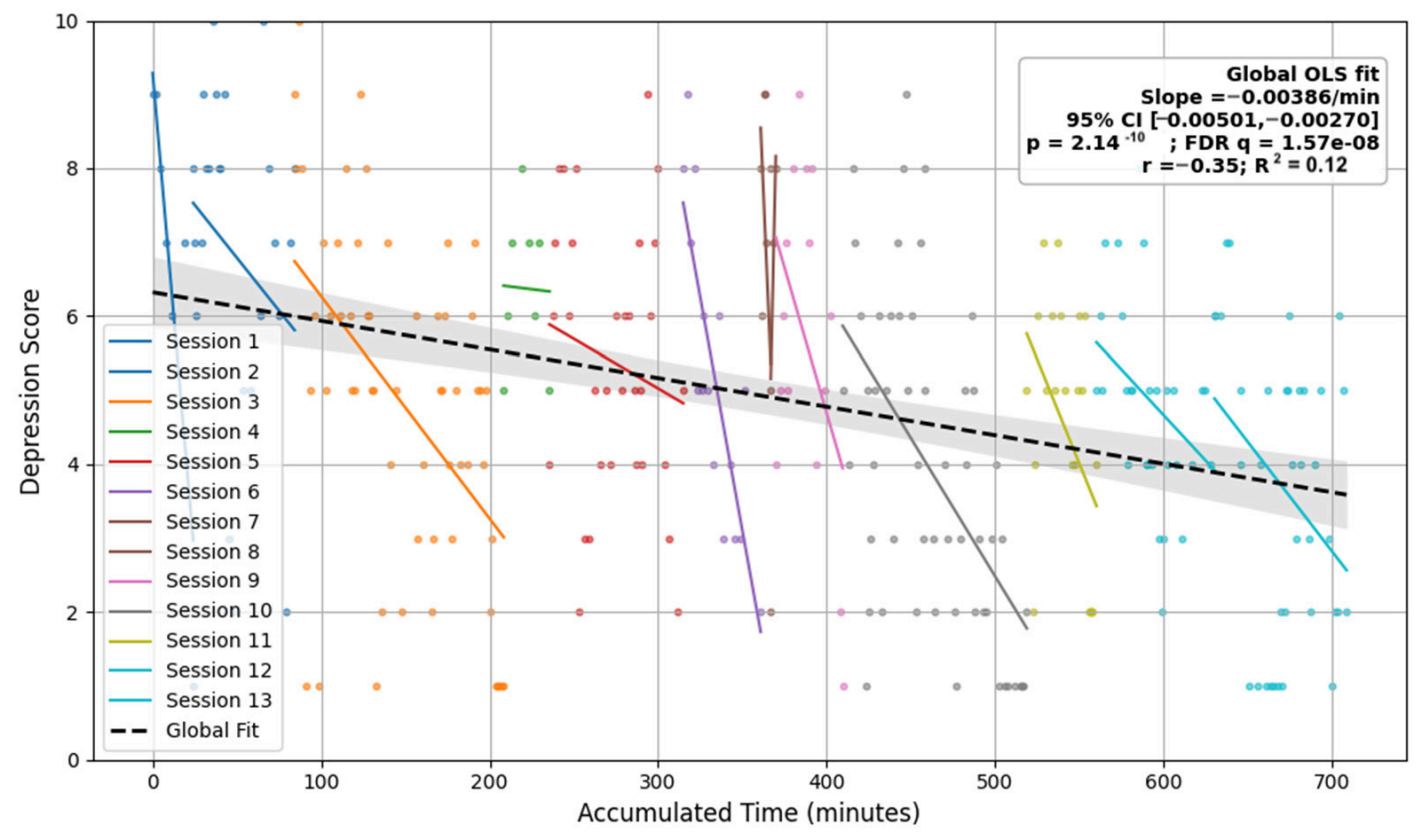
Visual example of a plot for a user who discussed ‘depression’ across multiple sessions. Colored dots represent individual message-level data points, and solid colored lines represent session-specific fitted trajectories. Colors are used to visually distinguish sessions; because the color palette repeats across the 13 sessions, the same color may be used for more than one session. The dashed black line is the global fit through all data points, with its statistics reported in the plot’s corner. The 95% confidence intervals are depicted in the gray shaded area, and the effect sizes are expressed as r and R^2^.

**Table 1:** Distribution of session counts per user for five conversation topics with an overall benchmark (“ALL”). The asterisk (*) denotes a significantly different topic compared to the aggregate mean according to a Mann–Whitney U test.

Topic	*n* of Session Count	Session Count (μ)	Standard Deviation (σ)	*p* Value
Suicidality	98	2.88	1.81	0.102
Work-related	1083	3.57	3.75	0.041 *
Grief	807	4.08	4.17	0.750
Loneliness	727	4.18	4.34	0.074
Depression	1384	4.37	5.31	0.345
ALL	*N* = 4099	4.00	4.43	

**Table 2:** User-level sentiment trajectory patterns in five topic–emotion pairs: depression|depression, grief|grief, loneliness|loneliness, suicidality|despair, and work-related|stress. The significance-adjusted ratio represents the percentage of all statistically significant trajectories that had a negative slope [significant negative/(significant negative + significant positive)]. The direction-adjusted ratio represents the percentage of all negative trajectories that reached a significant level [significant negative/(significant negative + non-significant negative)]. Individual trajectory significance was determined from the two-sided test of each user-specific OLS slope at *p* ≤ 0.05 and FDR correction is applied (Benjamini–Hochberg false discovery rate). Ratios are presented with 95% confidence intervals and are descriptive summaries. Statistical significance is used to classify individual OLS regression slopes.

Topic–Emotion Pair	Significance-Adjusted Ratio (%) (*p*-Value) (95% CI)	Direction-Adjusted Ratio (%) (*p*-Value) (95% CI)
Depression|depression	86.1 (1.98 × 10^−16^) (78.8–91.1)	44.7 (3.89 × 10^−69^) (38.5–51.1)
Grief|grief	85.7 (4.92 × 10^−15^) (77.4–91.3)	55.3 (1.89 × 10^−65^) (47.3–62.9)
Loneliness|loneliness	72.7 (1.98 × 10^−16^) (61.0–82.0)	44.0 (2.75 × 10^−33^) (35.1–53.4)
Suicidality|despair	90.9 (0.00586) (62.3–98.4)	40.0 (7.64 × 10^−8^) (23.4–59.3)
Work-related|stress	82.4 (4.87 × 10^−12^) (73.8–88.5)	43.3 (6.28 × 10^−51^) (36.5–50.3)

**Table 3:** Average time between sessions (in days) for users who returned to the DPS service, shown across five mental-health topics with an overall “ALL” benchmark for comparison. Asterisks indicate the Mann–Whitney U result versus the overall sample (*p* < 0.05: *, *p* < 0.001: ***).

Topic	Average Time Between Sessions (Days)	Standard Deviation (σ)	*p*-Value (Compared to Average)
Suicidality	18.6	28.1 ***	1.56 × 10^−6^
Work-related	10.8	30.1	0.651
Grief	9.4	25.8	0.550
Loneliness	11.8	29.8 *	0.034
Depression	9.6	28.4 *	0.012
ALL	10.4	28.6	

**Table 4:** Distribution of the number of resources that were shared during peer-chat sessions for each topic, alongside the overall average (marked “ALL”). Resource-count means and Mann–Whitney U tests were computed after topic-wise Tukey outlier removal (Q3 + 1.5 × IQR). Asterisks denote the Mann–Whitney U test result versus the aggregate sample (*p* < 0.05: *, *p* < 0.01: **).

Topic	Number of Resources per Session (μ)	Standard Deviation (σ)	*p*-Value (Compared to Average)
Suicidality	12.44	12.30 **	0.003
Work-related	9.48	10.58	0.729
Grief	8.29	9.17	0.087
Loneliness	10.44	11.44 *	0.043
Depression	8.59	9.37	0.250
ALL	9.19	10.17	

## Data Availability

The datasets generated and analyzed during the current study are not publicly available due to proprietary data. Data is available from the corresponding author upon reasonable request.

## Supplementary Materials

The following supporting information can be downloaded at https://www.mdpi.com/article/10.3390/jcm15197677/s1. Table S1: Distribution of sessions and users by conversation topic; Table S2: Racial and Ethnic Distribution of Users Discussing Each Topic (%). AI/AN: American Indian or Alaska Native, A: Asian, B: Black or African American, H: Hispanic or Latino, ME/NA: Middle Eastern or North African, NH/PI: Native Hawaiian or Pacific Islander, W: White; Table S3: Sensitivity analyses of longitudinal sentiment trajectory; Table S4: Distribution of the peer interaction counts: the number of distinct peers a participant engaged with during a single session. Topics (Depression, Work-related, Grief, Loneliness, and Suicidality) are examined. The bottom row aggregates all records (“ALL”) for comparison; Table S5: Session length for returning users across five mental-health topics, and overall average “ALL”. While most sessions lasted ~50 min, Suicidality sessions were significantly longer and had a higher variation due to more outliers and a smaller sample size. Asterisks indicate a Mann–Whitney U test against the aggregate distribution (*p* < 0.05). Textbox S1: Example AI prompt for NLP-based sentiment scoring.

## Abbreviations

The following abbreviations are used in this manuscript:

AI	Artificial Intelligence
BRFSS	Behavioral Risk Factor Surveillance System
CCPA	California Consumer Privacy Act
DPS	Digital Peer Support
GDPR	General Data Protection Regulation
NLP	Natural Language Processing
PHI	Protected Health Information
PII	Personally Identifiable Information
SAMHSA	Substance Abuse and Mental Health Services Administration
SDT	Self-Determination Theory
UTC	Coordinated Universal Time
