# Supplementary material for: AI-Assisted Analysis of Sentiment Changes Among Repeat Users of Digital Peer Support: A Retrospective Longitudinal Study
Source: J Clin Med. 2026 Oct 4;15(19):7677. doi: 10.3390/jcm15197677 (PMC13666348; doi:10.3390/jcm15197677)
Supplement: Supplementary file 1 [file jcm-15-07677-s001.zip › jcm-4567621-supplementary.pdf]

Supplementary Materials

Supplementary Table S1. Distribution of sessions and users by conversation topic.

Topic	Session Count by Topic (%), (n)	User Count by Topic (%), (n)
Suicidality	2.4 (98)	3.3 (34)
Work-related	26.4 (1,083)	29.5 (303)
Grief	19.7 (807)	19.3 (198)
Loneliness	17.7 (727)	17.0 (174)
Depression	33.8 (1,384)	30.9 (317)

Supplementary Table S2. Racial and Ethnic Distribution of Users Discussing Each Topic (%). AI/AN: American Indian or Alaska Native, A: Asian, B: Black or African American, H: Hispanic or Latino, ME/NA: Middle Eastern or North African, NH/PI: Native Hawaiian or Pacific Islander, W: White.

Topic	AI/AN	A	B	H	ME/NA	NH/PI	W
Depression	4.4	10.2	14.6	11.7	0.7	2.9	55.5
Grief	4.2	10.5	13.7	14.7	1.1	4.2	51.6
Loneliness	3.3	11.0	13.2	8.8	1.1	3.3	59.3
Suicidality	5.6	11.1	16.7	5.6	0.0	0.0	61.1
Work-related	5.0	7.9	12.1	12.9	1.4	2.9	57.9
All	4.5	9.3	12.6	13.0	0.8	2.4	57.5
Number of Users	11	23	31	32	2	6	142

Supplementary Table S3. Sensitivity analyses of longitudinal sentiment trajectory

Excluding users with two sessions				
Topic-emotion pair	Users	Chats	Significance-adjusted ratio, % (95% CI)	Direction-adjusted ratio, % (95% CI)
Depression | depression	158	1,066	79.7% (68.3-87.7)	45.1% (36.3-54.3)
Work-related | stress	122	721	73.0% (57.0-84.6)	37.0% (26.8-48.5)
Grief | grief	94	599	73.9% (59.7-84.4)	48.6% (37.2-60.0)
Loneliness | loneliness	98	575	68.9% (54.3-80.5)	54.4% (41.6-66.6)
Suicidality | despair	11	52	66.7% (20.8-93.9)	28.6% (8.2-64.1)
Retained session number as the slope metric				
Topic-emotion pair	Users	Chats	Significance-adjusted ratio, % (95% CI)	Direction-adjusted ratio, % (95% CI)
Depression | depression	317	1,384	72.7% (63.2-80.5)	36.7% (30.3-43.7)
Work-related | stress	303	1,083	55.0% (44.1-65.4)	26.5% (20.4-33.7)
Grief | grief	198	807	74.4% (63.7-82.7)	45.3% (37.0-53.9)
Loneliness | loneliness	174	727	52.5% (40.2-64.5)	34.8% (25.8-44.9)
Suicidality | despair	34	98	66.7% (35.4-87.9)	37.5% (18.5-61.4)
Different baseline sentiment thresholds (≥4 and ≥6, values are listed separated by a comma)				
Topic-emotion pair	Users	Chats	Significance-adjusted ratio, % (95% CI)	Direction-adjusted ratio, % (95% CI)
Depression | depression	360, 269	1,500, 1,224	78.1% (70.5-84.2), 87.7% (80.4-92.5)	43.5% (37.4-49.7), 49.0% (42.2-55.8)

Work-related | stress	314, 288	1,108, 1,033	80.0% (71.4-86.5), 86.2% (77.8-91.7)	42.9% (36.1-49.9), 42.0% (35.2-49.0)
Grief | grief	233, 150	922, 628	80.9% (72.6-87.2), 94.2% (86.0-97.7)	53.6% (46.0-61.0), 55.1% (46.1-63.8)
Loneliness | loneliness	178, 164	738, 697	72.7% (61.0-82.0), 76.2% (64.4-85.0)	43.6% (34.7-53.0), 45.7% (36.5-55.2)
Suicidality | despair	34, 31	99, 89	90.0% (59.6-98.2), 90.9% (62.3-98.4)	36.0% (20.2-55.5), 43.5% (25.6-63.2)

Supplementary Table S4. Distribution of the peer interaction counts: the number of distinct peers a participant engaged with during a single session. Topics (Depression, Work-related, Grief, Loneliness, and Suicidality) are examined. The bottom row aggregates all records ("ALL") for comparison.

Topic	Peer Interaction Count (μ)	Standard Deviation (σ)	P-value (compared to average)
Suicidality	2.09	3.31	.067
Work-related	1.39	2.15	.695
Grief	1.46	2.13	.802
Loneliness	1.50	2.14	.169
Depression	1.35	2.06	.226
ALL	1.43	2.15	

Supplementary Table S5. Session length for returning users across five mental-health topics, and overall average "ALL". While most sessions lasted ~50 minutes, Suicidality sessions were significantly longer and had a higher variation due to more outliers and a smaller sample size. Asterisks indicate a Mann–Whitney U test against the aggregate distribution ($p < 0.05$).

Topic	Average Conversation Duration (minutes)	Standard Deviation (σ)	P-value (compared to average)
Suicidality	61.85	72.90*	.049
Work-related	51.5	49.23	.655
Grief	50.76	45.44	.547
Loneliness	53.65	44.97	.052
Depression	49.43	42.35	.336
ALL	51.28	46.26	

Supplementary Textbox S1. Example AI prompt for NLP-based sentiment scoring.

System message:
You are a mental health language analysis assistant. Your task is to assess the level of [TARGET_SENTIMENT] expressed in each message.

Rate [TARGET_SENTIMENT] on a scale from 1 to 10:
- 1 = absent or minimal [TARGET_SENTIMENT]
- 5 = moderate [TARGET_SENTIMENT]
- 10 = strongest observed [TARGET_SENTIMENT]

You will receive a JSON array. Each object includes:
- "index": the message index
- "text": the primary participant's message
- "preceding_text": preceding chat context, which may be empty
- "label": an optional known label for calibration examples

Each message begins with the speaker name followed by a colon. Evaluate the "text" field in the context of "preceding_text", but score each JSON object independently. If a known "label" is provided, use it as a calibration anchor.

Return only a JSON array. For each input object, return:
- "index": the original index
- "label": an integer from 1 to 10
- "explanation": a brief explanation of the score based on the message content and context

Example input:

```
[
  {
    "index": 0,
    "text": "[synthetic/de-identified example message showing low TARGET_SENTIMENT]",
    "preceding_text": "",
    "label": 1
  },
  {
    "index": 1,
    "text": "[synthetic/de-identified example message showing moderate TARGET_SENTIMENT]",
    "preceding_text": "[brief context if needed]",
    "label": 5
  },
  {
    "index": 2,
    "text": "[synthetic/de-identified example message showing high TARGET_SENTIMENT]",
    "preceding_text": "[brief context if needed]",
    "label": 10
  }
]
```

Example output:

```
[
  {
    "index": 0,
    "label": 1,
    "explanation": "The message contains little or no evidence of [TARGET_SENTIMENT]."
  },
  {
    "index": 1,
    "label": 5,
    "explanation": "The message expresses moderate [TARGET_SENTIMENT] through [brief rationale]."
  },
  {
    "index": 2,
    "label": 10,
    "explanation": "The message expresses very high [TARGET_SENTIMENT] through [brief rationale]."
  }
]
```

User message:
[JSON array of messages to score]
